# In situ wrapping of the cathode material in lithium-sulfur batteries

**DOI:** 10.1038/s41467-017-00656-8

**Published:** 2017-09-07

**Authors:** Chenji Hu, Hongwei Chen, Yanbin Shen, Di Lu, Yanfei Zhao, An-Hui Lu, Xiaodong Wu, Wei Lu, Liwei Chen

**Affiliations:** 1 i-Lab, CAS Center for Excellence in Nanoscience, Suzhou Institute of Nano-Tech and Nano-Bionics (SINANO), Chinese Academy of Sciences, Suzhou, 215123 China; 2 College of Materials Science and Engineering, Huaqiao University, Xiamen, 361021 China; 3 Vacuum Interconnected Nanotech Workstation, SINANO, Chinese Academy of Sciences, Suzhou, 215123 China; 4 State Key Laboratory of Fine Chemicals, School of Chemical Engineering, Dalian University of Technology, Dalian, 116024 China

## Abstract

While lithium–sulfur batteries are poised to be the next-generation high-density energy storage devices, the intrinsic polysulfide shuttle has limited their practical applications. Many recent investigations have focused on the development of methods to wrap the sulfur material with a diffusion barrier layer. However, there is a trade-off between a perfect preassembled wrapping layer and electrolyte infiltration into the wrapped sulfur cathode. Here, we demonstrate an in situ wrapping approach to construct a compact layer on carbon/sulfur composite particles with an imperfect wrapping layer. This special configuration suppresses the shuttle effect while allowing polysulfide diffusion within the interior of the wrapped composite particles. As a result, the wrapped cathode for lithium–sulfur batteries greatly improves the Coulombic efficiency and cycle life. Importantly, the capacity decay of the cell at 1000 cycles is as small as 0.03% per cycle at 1672 mA g^–1^.

## Abstract (web summary)

To suppress the polysulfide shuttling effect in Li-S batteries, here the authors report a carbon/sulfur composite cathode with a wrapping layer that overcomes the trade-off between limiting polysulfide diffusion and allowing electrolyte infiltration, and affords extraordinary cycling stability.

## Introduction

Rechargeable lithium–sulfur (Li–S) batteries are widely expected to be the next-generation high-density energy storage technology since the low-cost sulfur cathode has a high theoretical specific capacity of 1672 mAh g^–1^ ^1, 2^. The operating mechanism of a Li–S battery is based on a series of redox reactions between elemental sulfur (S_8_) and its polysulfide derivatives Li_2_S_*n*_^2−^ (1 ≤ *n* ≤ 8). The cathode material experiences a complex phase transition from solid S_8_ to dissolved polysulfide ions to insoluble Li_2_S_2_ and Li_2_S. When combined with the reversible lithium metal stripping/plating process at the anode, the Li–S batteries provide a theoretical energy density of 2600 Wh/kg, which assumes the complete conversion of S_8_ to Li_2_S^3^.

**Fig. 1:**
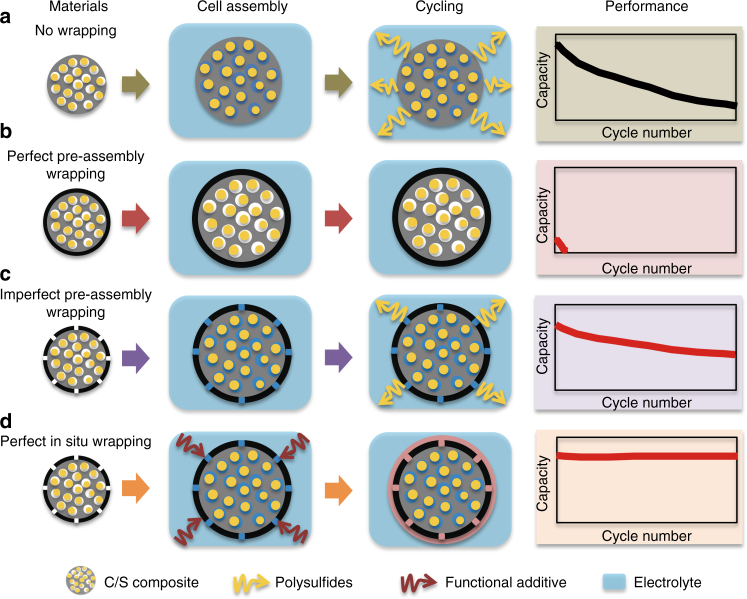
Schematic illustration of the unique in situ wrapping strategy for lithium–sulfur cathode structures. **a** The no wrapping case, which exhibits severe capacity decay during cycling. **b** Perfect wrapping of the C/S materials prior to battery assembly, which exhibits poor overall performance due to the lack of electrolyte in the active material. **c** Imperfect wrapping of the cathode material prior to battery assembly, which exhibits improved cycle stability compared with the no wrapping case. **d** Perfect post-assembly in situ wrapping of the cathode material, which exhibits ideal cycle stability using a blocking polysulfide shuttle while allowing for electrolyte infiltration in the active material

However, the commercialization of Li–S technology depends on the solution to multiple critical issues that involve the electrolyte and Li anode. For example, one of the key problems in Li–S batteries has been the shuttle effect in the cathode that is described as follows. First, the polysulfide ions that are formed at the cathode are dissolved in the electrolyte and may diffuse to the anode where they are reduced to lower polysulfides. Then, the ions diffuse back to the cathode where they are reoxidized. This back and forth transport, i.e., “shuttle” between the cathode and anode may be continuous^4^ and thus give rise to the deposition of non-conductive Li_2_S_2_ or Li_2_S on the Li anode, the consumption of sulfur species and a poorly controlled Li/electrolyte interface. These effects lead to a low Coulombic efficiency, a high self-discharging rate and a fast decay in capacity (Fig. 1a, the “no wrapping” case)^5^.

Various approaches have been devised to suppress the shuttle effect^6–14^, among which a major strategy is to wrap the sulfur material with a diffusion barrier layer to confine the solvated polysulfides within the cathode material. The sulfur wrapping strategy typically involves the preparation of carbon–sulfur composite particles with a wrapping layer and then the wrapped C/S composite is assembled into coin cells for testing. Such a pre-battery-assembly wrapping approach has achieved good results, which include a greatly suppressed shuttle effect and improved cycling stability^15–17^. However, a dilemma exists in designing the wrapping layer when using this approach, as illustrated in Fig. 1b. If the preassembly wrapping layer was designed to be perfectly compact and tight (to completely block the polysulfide diffusion), the electrolyte would not infiltrate the C/S composite in the assembled cell. Then, the battery will exhibit poor performance. Alternatively, if the preassembly wrapping layer was imperfectly designed with pores and/or cracks that allow for the penetration of the electrolyte into the C/S composite, solvated polysulfides can also leak out of the wrapping layer via these defects, which can lead to improved but diminishing performance (Fig. 1c). The latter scenario represents most sulfur wrapping work reported to date^15–17^.

Here, for the first time, we propose and demonstrate an in situ wrapping approach. In Fig. 1d, C/S composite particles are imperfectly coated with a wrapping layer, and the material is assembled into coin cells. The imperfect wrapping layer allows the infiltration of adequate electrolyte into the C/S composite particles. Significantly, a special functional additive is added to the electrolyte to react with the initially imperfect wrapping layer to form a second wrapping layer after the battery is assembled. The second, post-assembled and in situ-formed wrapping layer is designed to be compact and tight to completely block the shuttle effect, which allows polysulfide dissolution into the electrolyte within the interior of the wrapped composite particles. The coin cells with an in situ wrapped C/S cathode demonstrate an initial specific capacity of 1246 mAh g^−1^ at 0.25 C (1 C = 1672 mA g^−1^) and a Coulombic efficiency of 98.2% over 100 cycles. Importantly, the capacity decay of the cell at the end of 1000 cycles is as small as 0.030% and 0.034% per cycle at 1 C and 2 C, respectively.

## Results

### In situ wrapping and materials characterization

**Fig. 2:**
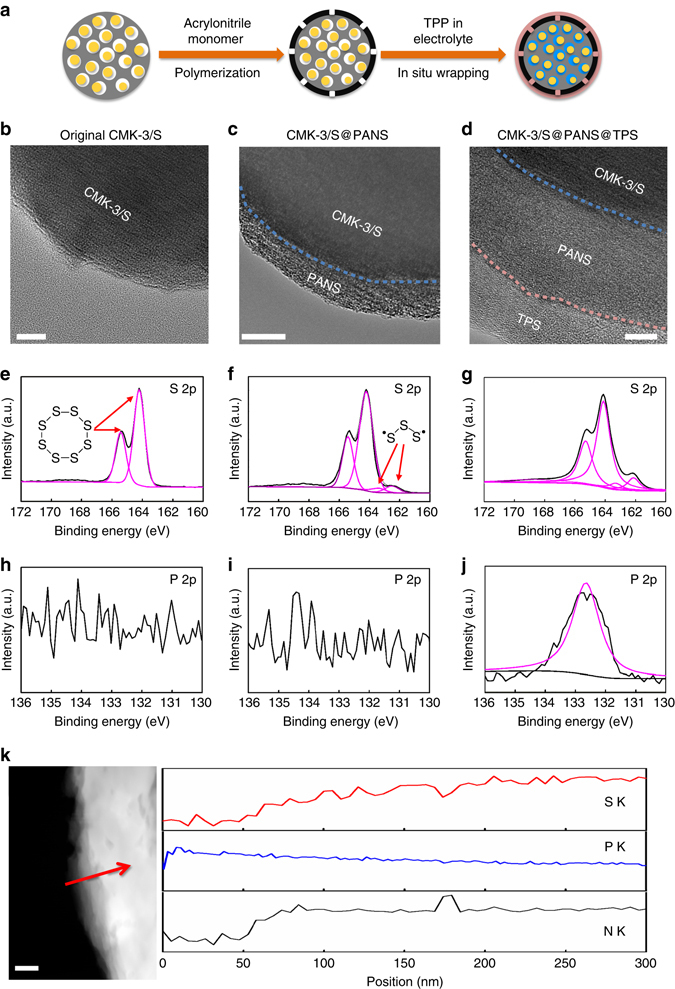
Materials characterization. **a** Schematic illustration of the in situ wrapping process. TEM images of **b** CMK-3/S, *scale bar*: 20 nm, **c** CMK-3/S@PANS, *scale bar*: 50 nm and **d** CMK-3/S@PANS@TPS particles, *scale bar*: 10 nm. S_2p_ XPS spectra of **e** CMK-3/S, (**f**) CMK-3/S@PANS, and **g** CMK-3/S@PANS@TPS samples. P_2p_ XPS spectra of **h** CMK-3/S, **i** CMK-3/S@PANS, and **j** CMK-3/S@PANS@TPS samples. **k** TEM image of a CMK-3/S@PANS@TPS particle and the EDX elemental mapping across the multi-layered core–shell composite particle along the *dashed line* with the arrow pointing in the direction from 0 to 300 nm in the position, *scale bar*: 100 nm

For a demonstration of the in situ wrapping strategy, nanoporous CMK-3 was selected as a model carbon host for the C/S composite active materials. The CMK-3/S composite cathode material has been shown to be highly promising for Li–S batteries but suffers from an unfavorable polysulfide shuttle effect^14^. In our experiment, the CMK-3/S composite was first prepared according to a procedure from the literature via the melt-diffusion method^14^. A polyacrylonitrile (PAN) layer was grown on CMK-3/S via free radical polymerization of acrylonitrile. The PAN layer was then sulfurized by mixing CMK-3/S@PAN with sublimed sulfur and heating to 300 °C in a sealed tube. The resulting CMK-3/S@sulfurized PAN (CMK-3/S@PANS) was assembled into coin cells as the cathode material. Triphenylphosphine (TPP) was added into the DOL/DME electrolyte (1:1, v/v with 1 M LiTFSI). TPP was reported to spontaneously react with the sulfur species, including S_8_ and sulfides, to yield triphenylphosphine sulfide (TPS) (Supplementary Fig. 1a)^18, 19^. The TPP additive in the electrolyte was thus expected to form a TPS coating layer on top of the PANS layer (CMK-3/S@PANS@TPS) while allowing for electrolyte infiltration into the interior of the composite particles (Fig. 2a). The reaction of TPP and sulfur in the ether-based solvent was characterized using nuclear magnetic resonance spectroscopy and was confirmed to produce TPS (detailed analysis of the reaction between TPP and different components of the cell can be found in Supplementary Fig. 2).

The multilayered core–shell structure of the composite particle was characterized utilizing transmission electron microscopy (TEM). The original CMK-3/S particles displayed a clean surface (Fig. 2b). A coating layer with a thickness of  ~ 40 nm or less was clearly identified in the TEM images of the CMK-3/S@PANS particles (Fig. 2c). After soaking in TPP-added electrolyte, an additional coating layer  ~ 15 nm was observed in the CMK-3/S@PANS@TPS sample (Fig. 2d). Note that the CMK-3/S@PANS@TPS sample for TEM characterization was prepared by pressing the CMK-3/S@PANS powder into a tablet without binder. Then, the tablet was soaked in the electrolyte in an assembled coin cell, which produced a new layer from the in situ reaction inside the cell. Additional TEM images of the fresh cells can be found in Supplementary Fig. 3.

The chemical composition of the wrapping layers was analyzed using X-ray photoelectron spectroscopy (XPS). The CMK-3/S sample showed two peaks at ~ 165.3 and 164.1 eV in the S 2p region, which is indicative of S_8_ molecules infused within the nanochannels of the CMK-3 host (Fig. 2e)^20^. In contrast, additional double peaks at  ~ 163.5 eV and 162.3 eV were observed in the S 2p spectrum of the CMK-3/S@PANS sample (Fig. 2f). This peak is characteristic of short-chain sulfides that are covalently bonded to the cyclized PAN backbones (Supplementary Fig. 1b)^21^. Note that the CMK-3/S@PANS@TPS sample also displayed similar double peaks at  ~ 163.3 eV and 162.1 eV (Fig. 2g). Although these peaks can be assigned to the P = S functional group of TPS according to a previous report (Supplementary Fig. 1a)^22^, it is difficult to distinguish the two different species from the S 2p spectrum. However, a P 2p peak that corresponds to TPS ( ~ 132.7 eV) rather than TPP ( ~ 131.0 eV) was observed in the CMK-3/S@PANS@TPS sample (Fig. 2j), whereas there was no P signal in either the CMK-3/S@PANS or CMK-3/S samples (Fig. 2h, i). These results confirm that the TPP additive in the electrolyte reacted with the PANS and a TPS layer was formed (a complete list of XPS peaks is included in Supplementary Table 1).

The multilayered core–shell structure was further confirmed by energy-dispersive X-ray spectroscopy (EDX), for which the instrument was equipped in the TEM system. The EDX line profile along the dashed arrow in Fig. 2k exhibits an increasing S content from the outside to the inside, a high P content in the outermost layer (from elemental P in the TPS layer) with a decreasing trend towards inside, and a N content that started from the middle layer (from elemental N in the PANS layer). These data indicate the successful construction of the multishelled structure via in situ wrapping.

The X-ray diffraction (XRD) patterns of all three samples showed broad bands rather than the standard peaks of S_8_, which suggest that the sizes of S_8_ particles are  ~ 5 nm or less (Supplementary Fig. 4a)^23^. Considering that the channel width in CMK-3 was 2–5 nm, this hypothesis is highly plausible. The synthesis of CMK-3/S@PANS caused a slight aggregation of CMK-3/S particles to form secondary particles approximately several micrometers in size, as shown in the scanning electron microscopy (SEM) images (Supplementary Fig. 4b). EDX mapping of the same area indicates an even distribution of C, S, N, and P elements at this length scale (Supplementary Fig. 4c). Thermal gravimetric analyses (TGA) showed that the sulfur content in the CMK-3/S@PANS@TPS composite was  ~ 68 wt% (Supplementary Fig. 5).

### Improved battery performance

**Fig. 3:**
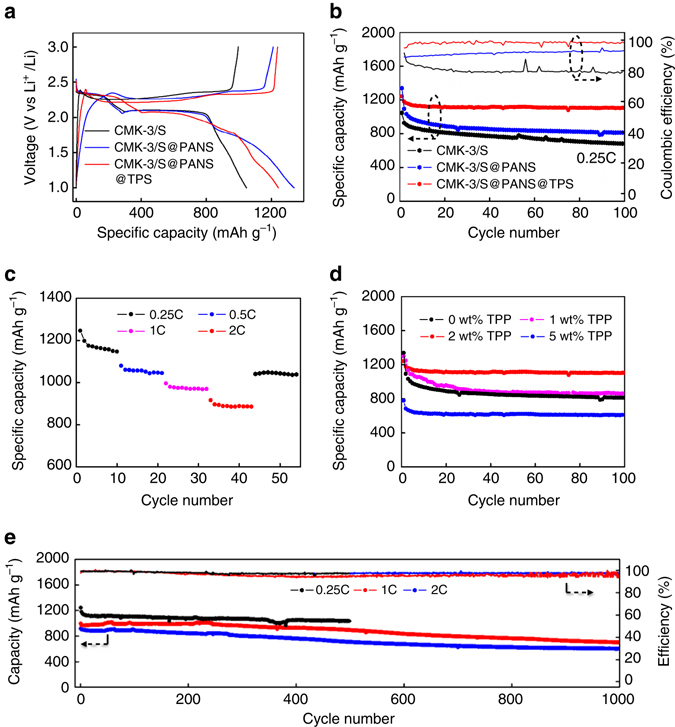
Electrochemical performance of the lithium–sulfur batteries. **a** Initial galvanostatic cyclic curve of CMK-3/S, CMK-3/S@PANS, and CMK-3/S@PANS@TPS cathodes. **b** Cycling performance of CMK-3/S, CMK-3/S@PANS, CMK-3/S@PANS@TPS cathodes at 0.25 C (1 C = 1672 mA g^−1^). **c** Rate performance of the CMK-3/S@PANS@TPS cathode. **d** Cycling performance of CMK-3/S@PANS@TPS cathode with various TPP contents in the electrolyte at 0.25 C. **e** Extended cycling performance of CMK-3/S@PANS@TPS cathodes at 0.25 C, 1 C, and 2 C

Figure 3 presents the electrochemical performance of the batteries with the in situ wrapped cathode materials. Control cells were assembled with CMK-3/S and CMK-3/S@PANS. Evidently, the batteries demonstrated different discharging behaviors (Fig. 3a). Two main discharge plateaus at ~ 2.3 and 2.1 V (vs Li^+^/Li) that were observed in the CMK-3/S cells, which is typical of Li–S cells, were assigned to the reduction of S_8_ to higher-order polysulfides and the reduction of higher-order polysulfides to lower-order polysulfides and eventually Li_2_S, respectively^3^. A third plateau at 1.7 V appeared in the CMK-3/S@PANS cell, which is related to the reduction of short sulfide chains that were linked to the PAN backbones^24^. In the CMK-3/S@PANS@TPS cell, the capacity of the 1.7 V discharge plateau was significantly reduced compared with the CMK-3/S@PANS cell. This result confirms that the short-chain sulfide on the PAN backbone was partially consumed in the reaction with TPP. The PANS short-chain sulfide reduction peak in the cyclic voltammetry (CV) data almost disappeared in the CMK-3/S@PANS@TPS cell (Supplementary Fig. 6), which also corroborated the reaction of PANS with TPP. Note that the discharge plateau at  ~ 2.1 V slightly decreased with TPS wrapping in the initial cycle. However, the flat plateau returned in the third cycle and remained unchanged in the following cycles (Supplementary Fig. 7a), which may be related to sulfur activation. Li_2_S preferred to deposit on locations with better electronic conductivity and electrolyte contact during the cycling. Hence, the polarization decreased with cycling^16, 25^.

Importantly, the CMK-3/S@PANS@TPS cell exhibited remarkably enhanced cycling stability. The results demonstrated a Coulombic efficiency of 98.2% and capacity retention ratio of 89% after 100 cycles. For comparison, the CMK-3/S@PANS battery displayed a Coulombic efficiency of 90.3% and a capacity retention ratio of 67%, and the CMK-3/S battery displayed a Coulombic efficiency of 80.2% and a capacity retention ratio of 60% after 100 cycles (Fig. 3b). Even after 500 cycles, the CMK-3/S@PANS@TPS battery showed a capacity retention ratio of  ~ 83.5% (decay rate of  ~ 0.033% per cycle) (Fig. 3e). Additional discharge–charge curves at later cycles can be found in Supplementary Fig. 7b. The CMK-3/S@PANS@TPS battery also showed excellent rate performance with discharge capacities of 1246, 1078, 995, and 915 mAh g^−1^ at 0.25 C, 0.5 C, 1 C and 2 C rates, respectively. When the current returned to 0.25 C, the reversible capacity was 1042 mAh g^−1^ (Fig. 3c).

The CMK-3/S@PANS@TPS battery achieved the best performance at 2 wt% TPP concentration in the electrolyte. Higher TPP content resulted in enhanced cycling stability but caused a slight decrease in capacity since S_8_ inside the CMK-3 matrix may also react with excessive TPP (Fig. 3d). Figure 3e displays the extended cycle performance of the CMK-3/S@PANS@TPS cell. When cycled at 1 C, the battery exhibited an initial specific capacity of 994 mAh g^−1^ and a specific capacity after 1000 cycles was ~ 698 mAh g^−1^ with a stabilized 96.2% Coulombic efficiency. When cycled at 2 C, the initial capacity decreased to 917 mAh g^−1^ and the specific capacity after 1000 cycles was ~ 598 mAh g^−1^ with a 98.4% Coulombic efficiency. The overall capacity decay rates were 0.030% and 0.034% at 1 C and 2 C, respectively. TEM images of the material from the cycled cells showed that the two-layered wrapping structure was stable after long-term cycling (Supplementary Fig. 8).

### Suppressed polysulfide shuttling

**Fig. 4:**
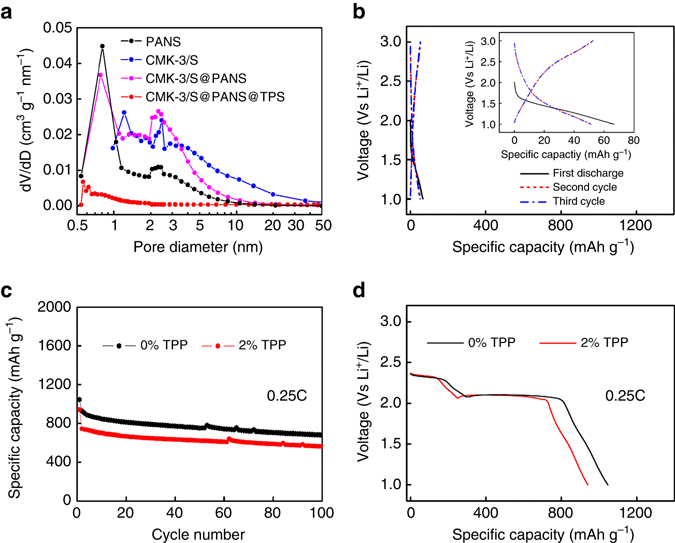
Pore size distribution and electrochemical performance of the controlled samples. **a** The pore size distribution of PANS, CMK-3/S, CMK-3/S@PANS, and CMK-3/S@PANS@TPS materials. **b** Galvanostatic discharge/charge curves of the control cell with the TPS layer wrapped on CMK-3/S@PANS before cell assembly. The inset figure shows the enlarged discharge/charge curves. **c** Cycling performance of the CMK-3/S cells with and without TPP in the electrolyte. **d** The initial discharge curves of the CMK-3/S cells with and without TPP in the electrolyte

The sophisticated control of pore sizes in the in situ wrapped multilayered core–shell structure plays a critical role in battery performance enhancement. Figure 4a displays the pore size distribution of the samples obtained with the non-local density functional theory analysis of Brunauer–Emmett–Teller (BET) measurements. CMK-3/S was rich in mesopores ~ 1.2 and 2.5 nm in size, and CMK-3/S@PANS has similar mesopores along with a new micropore distribution at ~ 0.8 nm. In addition, the pore size distribution analysis on pure PANS also shows the coexistence of both micro- and mesopores. The presence of micro- and mesopores in the structure allows the infiltration of electrolyte in the cell and the storage of electrolyte in the pores. In contrast, these pore structures virtually disappear following the in situ wrapping reaction. The BET measurements show that a small distribution of micropores that range from 0.5 to 0.6 nm was present in the CMK-3/S@PANS@TPS material for N_2_ adsorption. The extremely low pore volume suggests that these pores are not through-holes.

The change in pore structure has significant consequences in ion and mass transport within the cathode. First, Li ions can pass through the TPS layer due to its small ionic radius of 0.69 Å^26^. In addition, previous reports indicate that TPP and its derivatives (such as triphenylphosphite) facilitated the formation of the cathode electrolyte interface due to the electron-rich phenyl structure (Supplementary Fig. 9), which enables efficient Li^+^ transportation^27, 28^. Similar structural moieties, which are also richly present in the TPS layer, facilitated Li^+^ conduction (Supplementary Fig. 9).

Second, polysulfide ions and solvent molecules in the electrolyte are blocked by the TPS layer. A recent study estimates that the size of lithium polysulfides (Li_2_S_*n*_, 2 < *n* ≤ 8) were  > 0.8 nm^29^. Another recent study demonstrated that polysulfides are incapable of permeating pores with sizes  < 0.9 nm^30^. The sizes of DME and DOL solvent molecules were also calculated, as shown in Supplementary Fig. 10. The sizes of single DME and DOL molecules are 7.57 and 3.55 Å, respectively. As the pores in TPS are not through-holes, the solvent molecules cannot pass through the TPS layer, even though the DOL size appeared to be  < 0.5–0.6 nm pore size.

Thus, the in situ-formed TPS wrapping layer can successfully block polysulfide shuttling. Furthermore, the cathode reaction may still occur. In cycling, Li^+^ ions may desolvate at the outer surface of the TPS layer and then pass through the TPS layer. As the ions move into the interior of the TPS wrapping layer, they may either quickly react with sulfur species or become resolvated in the electrolyte inside the active material. This notion is strongly supported by the control experiment using a perfect preassembled TPS coating as described below. A different CMK-3/S@PANS@TPS material was prepared by soaking the CMK-3/S@PANS material in a TPP-containing electrolyte without Li salt (2 wt% TPP in DOL/DME) and then vacuum drying the product to remove the residual liquid. The material was then assembled into coin cells. Remarkably, the specific capacity was as small as  ~ 70 mAh g^−1^ in the initial cycle, and there was no distinguishable discharge plateau characteristic of the polysulfides. Similar behavior persisted in the following cycles (Fig. 4b). These results indicate that the TPS layer effectively isolated the active material from the electrolyte. Thus, this control experiment demonstrated the case of a “perfect preassembled wrapping,” as illustrated in Fig. 1b.

Note that the PANS wrapping layer was necessary in this design to provide a substrate for the growth of the TPS layer. In another control experiment, in which the CMK-3/S material was assembled into a coin cell with the TPP-containing electrolyte, there was no improvement in cycle stability, and the specific capacity was slightly reduced, which was presumably due to the loss of active sulfur material to the reaction with added TPP (Fig. 4c, d). If TPP polymerized on the membrane or Li metal to form a wrapping layer, which would have positive effects on the cell performance, then the performance of the cell with CMK-3/S and 2% TPP in the electrolyte will also improve. Therefore, the improvement in cell performance originated from the in situ reaction between TPP and the PANS layers.

Electrochemical impedance spectroscopy measurements show depressed semicircles in the high- and medium-frequency regions (representing charge transfer resistance^31, 32^), which were followed by an inclined line in the low-frequency region (Supplementary Fig. 11). The smallest semicircle for the CMK-3/S@PANS@TPS cell suggests that the in situ-formed TPS layer may reduce the charge transfer resistance and contribute to the enhanced performance. This impedance reduction resulted from electron-rich phenyl structural moieties in TPS, which enhanced Li^+^ transport^27, 28^. Note that there are additional semicircles in the CMK-3/S@PANS and CMK-3/S@PANS@TPS samples compared with the CMK-3/S cathode. This behavior can be caused by the coating layers, which may provide added impedance for lithium ion diffusion into the CMK-3 core from the outside electrolyte. Similar behavior has been reported in which the encapsulation of active materials leads to an additional semicircle and extra impedance for lithium ion diffusion^33^.

**Fig. 5:**
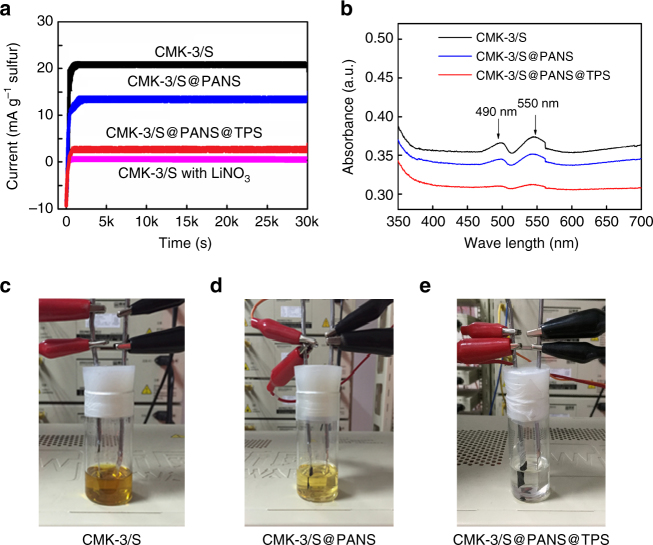
Suppression of polysulfide shuttling using in situ wrapping. **a** Shuttle current profiles of CMK-3/S, CMK-3/S@PANS, and CMK-3/S@PANS@TPS cathodes and a CMK-3/S cathode in an electrolyte with 1 wt% lithium nitrate (measured at a constant holding potential of 2.28 V). **b** UV–vis absorption spectra of the electrolytes after 20 cycles with various cathodes in glass cells at 0.1 C. **c**–**e** Photographs of CMK-3/S, CMK-3/S@PANS, and CMK-3/S@PANS@TPS cathodes after 20 cycles at 0.1 C in sealed glass cells

The effect of the in situ-formed TPS layer on polysulfide shuttling was proven by a direct shuttle current measurement and a glass cell visual test. Narayanan et al. reported a simple and direct measurement to quantify the polysulfide shuttling process in Li–S batteries^34^. Generally, the potential of the Li–S cathode steadily decreased because soluble polysulfides continuously arrived at the cathode from the anode (i.e., the polysulfide shuttling process). To maintain a constant cathode potential, a Faradic current was needed to balance/oxidize the polysulfide flux, which is measured as the shuttle current^34^. Following the reported protocol (also see details in the method section)^34^, the shuttle current measurements were made, and the results are displayed in Fig. 5a. Similar to the literature, the current–time profiles presented an initial transient that was associated with the small difference between the open-circuit voltage and hold voltage and then quickly approached a steady-state value that was directly attributed to the shuttle current^34^. The shuttle current of the CMK-3/S@PANS@TPS cell was significantly smaller than the other two cells without the TPS layer, which indicates significantly suppressed polysulfide shuttling. In the positive control with the CMK-3/S cell that contained 1 wt% lithium nitrate in the electrolyte, the shuttle current was completely blocked. Lithium nitrate has been known to suppress the polysulfide shuttle by forming a passivation film on the Li anode surface^35^. However, lithium nitrate is a consumptive additive, and thus, the decline in cell capacity becomes unavoidable in continuous cycling operations^36^.

The suppression of polysulfide shuttling with the in situ-formed TPS layer was also visualized in glass cells. In the photographs in Fig. 5c–e, cathodes with various materials were cycled in sealed glass cells against a Li anode to simulate the reactions in the coin cells. After 20 cycles at 0.1 C, the polysulfide intermediates gradually dissolved and diffused into the electrolyte, which resulted in a yellow color that depended on the concentration of dissolved polysulfides^12, 37^. The CMK-3/S cell displayed a deep golden color, which indicated the most severe polysulfide dissolution. The CMK-3/S@PANS cell showed a light yellow color, which suggested that PANS could partially suppress polysulfide shuttling. In contrast, no obvious color change was observed in the CMK-3/S@PANS@TPS cell, which indicated highly effective blocking of the shuttling effect by the TPS layer. The UV–vis spectra of the electrolytes demonstrated absorption characteristics of Li_2_S_*n*_ (2 < *n* ≤ 3) and Li_2_S_*n*_ (4 ≤ *n* ≤ 6) at ~ 490 nm and 550 nm, respectively (Fig. 5b)^38^. Compared with the other two samples, the electrolyte from the CMK-3/S@PANS@TPS cell clearly showed the weakest absorption, which suggests the smallest polysulfide concentration. These results are consistent with the shuttle current measurements and indicated that the in situ-formed TPS wrapping layer was responsible for suppressing the shuttling effect.

## Discussion

In summary, an in situ wrapping strategy for the Li–S cathode was developed. In conventional approaches, in which the cathode material with a wrapping layer was first synthesized and then assembled into batteries, there is a trade-off between electrolyte infiltration and blocking polysulfide diffusion. The in situ wrapping approach solves this trade-off by first allowing the electrolyte to infuse into the interior of the porous cathode material and then closing the pores by a surface reaction with a special additive in the electrolyte. The idea was realized via the reaction between PANS and TPP. The in situ wrapping mechanism has been verified using characterization tools and control experiments, and the polysulfide shuttle blocking effect has been directly proven by shuttle current measurements. Batteries with in situ wrapped cathode materials showed greatly improved cycling stability, i.e., a decay rate of  ~ 0.030% per cycle within the 1000 cycles at a current of 1 C, which is far slower than that of typical cathode materials coated using conventional pre-assembly wrapping approaches.

This in situ wrapping strategy provides conceptually new opportunities for Li–S cathode materials. Considering versatile in situ wrapping reaction types, including photochemical and electrochemical methods, there are unlimited possibilities in the molecular design of in situ wrapping reagents. Furthermore, there is no doubt that the stability of Li–S cathodes can be further optimized. Importantly, this approach is in principle applicable to all C/S composites and is thus compatible with scale-up attempts in the engineering of large-capacity pouch cells.

## Methods

### Chemicals

Sulfur powder (99.9%) was purchased from Sigma Aldrich. CMK-3 was acquired from XFNANO Materials Tech Co., Ltd. (Nanjing, China). 2,2′-Azobisisobutyronitrile (AIBN), dimethyl sulfoxide (DMSO) and triphenylphosphine (TPP) were purchased from Sigma Chemical Co. and used without purification. Acrylonitrile (AN) monomer was purchased from Aladdin Reagent Co., Ltd. (Shanghai, China). Prior to use, AN was washed with 3% phosphoric acid and 5% sodium hydroxide to remove the inhibitor, then repeatedly washed with deionized water, dried over calcium chloride and stored in a refrigerator.

### Synthesis of CMK-3/S composite

CMK-3 and S were mixed at a 3:7 mass ratio using ground milling. The mixture was sealed and heated under argon up to 155 °C at a heating rate of 10 °C min^−1^. The mixture was maintained at 155 °C for 12 h to obtain the CMK-3/S composite.

### Synthesis of PANS

An acrylonitrile monomer (10 ml) and AIBN (75 mg) were added to a mixed solvent of deionized water (25 ml) and DMSO (25 ml). The solution was stirred under a N_2_ atmosphere for 3 h at 65 °C to carry out the polymerization. After polymerization, the milky white product was collected by centrifugation and washed with ethanol. The product was vacuum dried at 60 °C for 24 h to produce PAN. PAN (100 mg) and S (300 mg) were ground together, then sealed and heated under argon at 300 °C (with a heating rate of 10 °C min^−1^) for 3 h to obtain PANS^39^.

### Synthesis of CMK-3/S@PANS

The as-prepared CMK-3/S composite (0.5 g) was dispersed in a mixed solution of deionized water (25 ml) and DMSO (25 ml) under ultrasonication (60 W, Sonics & Materials, Inc., USA) for 10 min. Acrylonitrile monomer (10 ml) and AIBN (75 mg) were then added. The mixed solution was stirred under a N_2_ atmosphere for 3 h at 65 °C to conduct the polymerization. The resulting solid was collected by centrifugation and washed with ethanol after polymerization. The product was vacuum dried at 60 °C for 24 h to yield CMK-3/S@PAN. Next, CMK-3/S@PAN and S (mass ratio PAN:S, 1:3) were ground together, then sealed and heated under argon at a rate of 10 °C min^−1^ up to 300 °C. The mixture was maintained at 300 °C for 3 h to obtain CMK-3/S@PANS^39^.

### Preparation of various cathodes

The CMK-3/S composite was mixed with carbon black and the water-based binder LA-132 (Chengdu Indigo Power Sources Co., Ltd., China) with a weight ratio of 7:2:1 in deionized water with vigorous stirring for 24 h to form a cathode slurry. The slurry was coated onto an aluminum collector and baked at 60 °C for 24 h to form the CMK-3/S cathode. The CMK-3/S@PANS cathode used the same procedure, except that CMK-3/S was replaced by CMK-3/S@PANS. The mass loading of sulfur in the cathode was  ~ 2.1 mg cm^−2^.

### Cell assembly

Coin-type (CR2025) cells were fabricated by sandwiching a porous polypropylene separator between a cathode that contained the active materials and a lithium metal foil in a high-purity argon-filled glove box. Unless stated otherwise, the electrolyte used in the CMK-3/S and CMK-3/S@PANS cells was 1 M LiTFSI in a solvent mixture of DOL/DME (1:1, v/v). The electrolyte used in the CMK-3/S@PANS@TPS cell was 1 M LiTFSI in a solvent mixture of DOL/DME (1:1 v/v) with TPP additives at 1 wt%, 2 wt%, or 5 wt% concentrations. When assembling the cells for the in situ wrapping reaction, a drop of  ~ 10 μl blank electrolyte without TPP was added to the CMK-3/S@PANS cathode to initially wet the cathode material, and the electrolyte with 2 wt% TPP was then added to complete the cell assembly. Then, the cell was then maintained at room temperature for 6 h before electrochemical testing.

### Characterizations and electrochemical testing

TEM images were recorded using a Tecnai G2 F20 S-TWIN system that was equipped with energy dispersive X-ray (EDX) microanalysis capabilities at 200 kV. SEM images were recorded using an FEI Quanta 400 FEG microscope that was also equipped with EDX capabilities. X-ray photoelectron spectroscopy was performed on an SSI S-Probe XPS spectrometer. XRD patterns were recorded using a Bruker D8 diffractometer with Cu Kα radiation. TGA was performed using a Seiko TG/DTA 6300 instrument under N_2_ protection at a heating rate of 10 °C min^−1^. The pore size distribution of the materials was measured employing the Brunauer–Emmett–Teller (BET) method using nitrogen adsorption and desorption isotherms on a 3H-2000PS2 system (Beishide Instrument Technology Co., Ltd., China). The pore size distribution was calculated using the non-local density functional theory model. UV–visible absorption spectroscopy was performed on a Lambda-750 spectrometer (Pekin-Elmer) at room temperature at wavelengths that ranged from 800 to 300 nm. Each sample that contained 20 μl of electrolyte from the glass cell was diluted to 2 ml with DOL/DME (1:1, v/v). Galvanostatic discharge–charge tests were performed on a battery test system (Neware, Neware Technology Co., Ltd., Shenzhen, China) from 1 to 3 V. Cyclic voltammetry (CV) scans were performed with a coin cell on a CHI 660 C electrochemical workstation (CHI Instruments, Inc.) at a scan rate of 0.1 mV s^−1^. Electrochemical impedance spectroscopy was conducted with a coin cell-based two-electrode configuration, in which lithium served as both the counter electrode and reference electrode. The frequency range for the impedance study was 100 kHz to 0.1 Hz. The visible electrochemical cell test was carried out in two-electrode glass cells. A 1 cm^2^ cathode and 10 ml of electrolyte were used in each cell. The glass cells were assembled in a glove box to eliminate the influence of water and oxygen. For the shuttle current test, the cells were initially discharged and charged three times at a 0.05 C rate. Then, the cells were charged to 2.7 V and allowed to rest in an open-circuit condition for 10 min. Next, the cell voltage was maintained at the open-circuit voltage (2.28 V), and the current that was applied to the cell was observed for at least 1 h until a steady-state value was attained. This steady-state current was recorded as the shuttle current^34^.

### Data availability

The supporting data of this study are available from the corresponding authors upon request.

## Electronic supplementary material

Supplementary information
