# Supplementary material for: In situ wrapping of the cathode material in lithium-sulfur batteries
Source: Nat Commun. 2017 Sep 7;8:479. doi: 10.1038/s41467-017-00656-8 (PMC5589852; doi:10.1038/s41467-017-00656-8)
Supplement: Supplementary file 1 — Supplementary information [file 41467_2017_656_MOESM1_ESM.pdf]

Description of Supplementary Files

File name: Supplementary Information
Description: Supplementary figures, supplementary table 1, supplementary notes and supplementary references.

Supplementary Figures

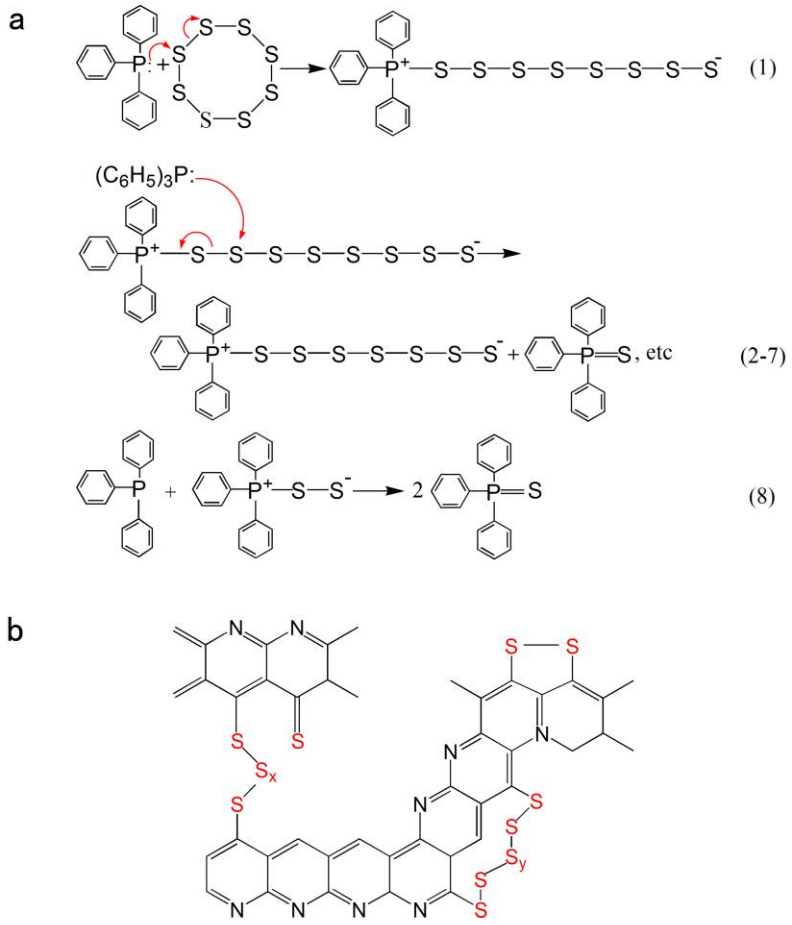

Supplementary Figure 1 | Proposed chemical structure and reaction.

(a) Spontaneous reactions between sulphur species and TPP, adopted from supplementary ref. 1. The reaction begins as a nucleophilic displacement of S on S_8 by the basic phosphine, which opens the sulphur ring to form a dipolar ion, then reacts rapidly in a series of follow-up reactions with more TPP, and the final resultant is $(C_6H_5)_3P{=}S$ (abbreviated as TPS).[1,2] **(b)** Proposed chemical structure of sulphurized PAN ($0 < x < 6$; $y = 1,2$) adopted from Supplementary ref. 2. The short polysulphide chains are covalently bonded to the cyclized and dehydrogenated PAN backbones through the C–S bonds.[3]

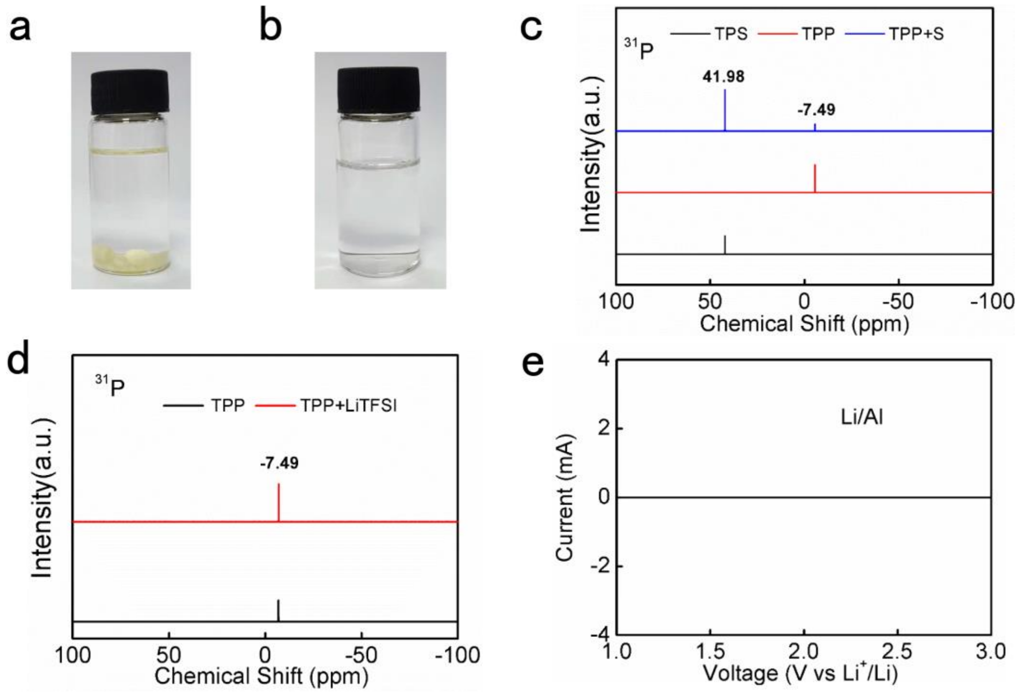

Supplementary Figure 2 | Reaction of triphenylphosphine and sulphur in the ether-based solvent. The photographs of **(a)** pristine state of sulphur in TPP solution and **(b)** after stay for 3 hours. **(c)** The P spectrum of NMR of TPP , TPS and the product of TPP react with S in DOL/DME. **(d)** The ^{31}P spectrum of TPP and TPP+LiTFSI. **(e)** the CV curves of Li||Al battery.

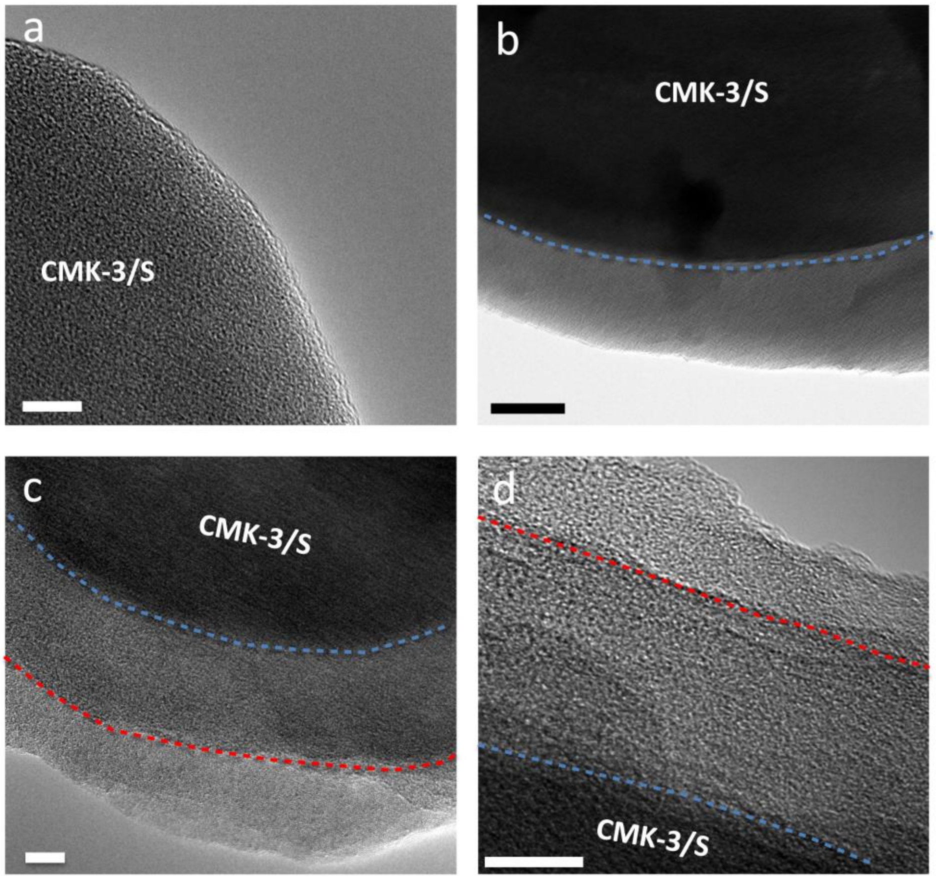

Supplementary Figure 3 | More TEM images of various cathode materials.

(a) CMK-3/S, scale bar = 10 nm , **(b)** CMK-3/S@PANS, scale bar = 50 nm and **(c-d)** CMK-3/S@PANS@TPS, scale bar = 10 nm.

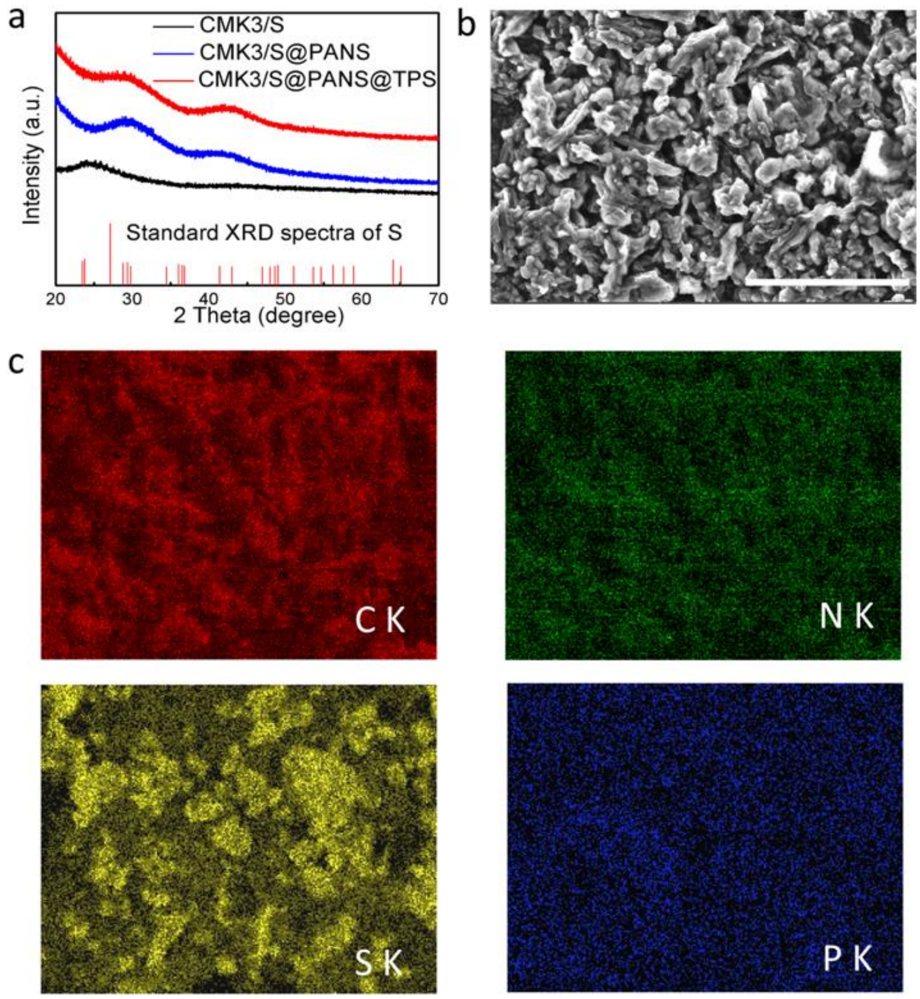

Supplementary Figure 4 | XRD, SEM and elemental mapping characterization.

(a) XRD patterns of CMK-3/S, CMK-3/S@PANS and CMK-3/S@PANS@TPS.

(b) SEM image of CMK-3/S@PANS@TPS, scale bar =20 μm. **(c)** Corresponding C, N, S and P elemental mapping in **(b)**.

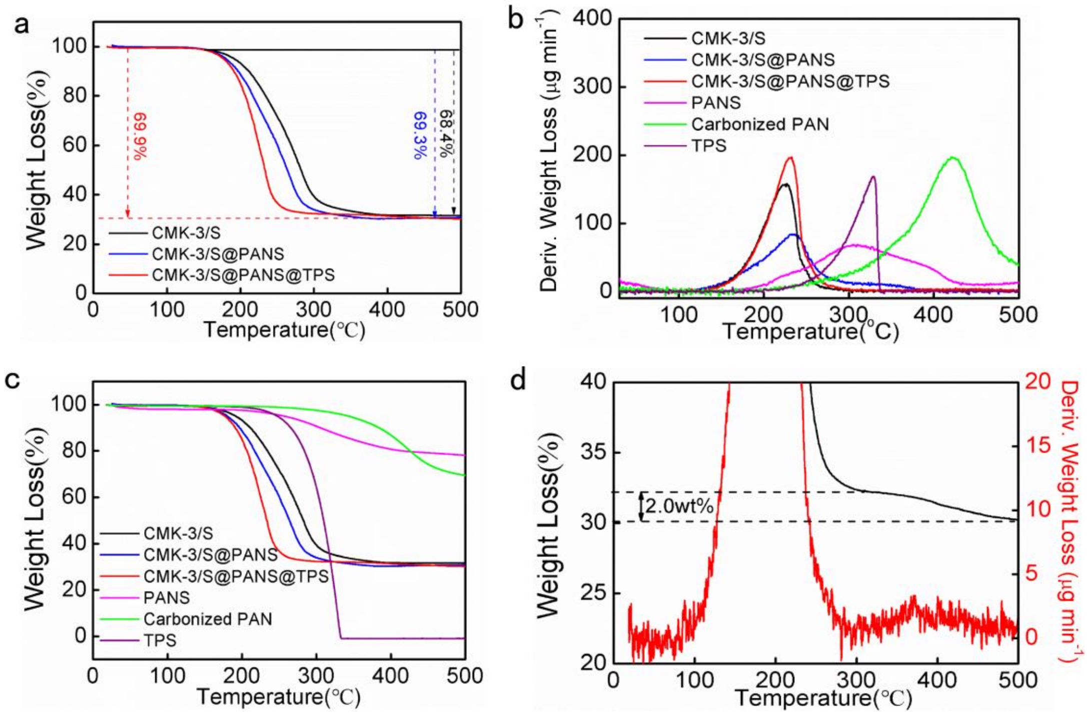

Supplementary Figure 5 | Thermal gravimetric and derivative thermogravimetric analysis. (a) TG curves of CMK-3/S, CMK-3/S@PANS and CMK-3/S@PANS@TPS. **(b)** The derivative thermogravimetric (DTG) analysis and **(c)** TG curves of different materials. **(d)** Enlarged TG and DTG curves of CMK-3/S@PANS@TPS showing the small weight loss in the 300-500 ^{o}C range.

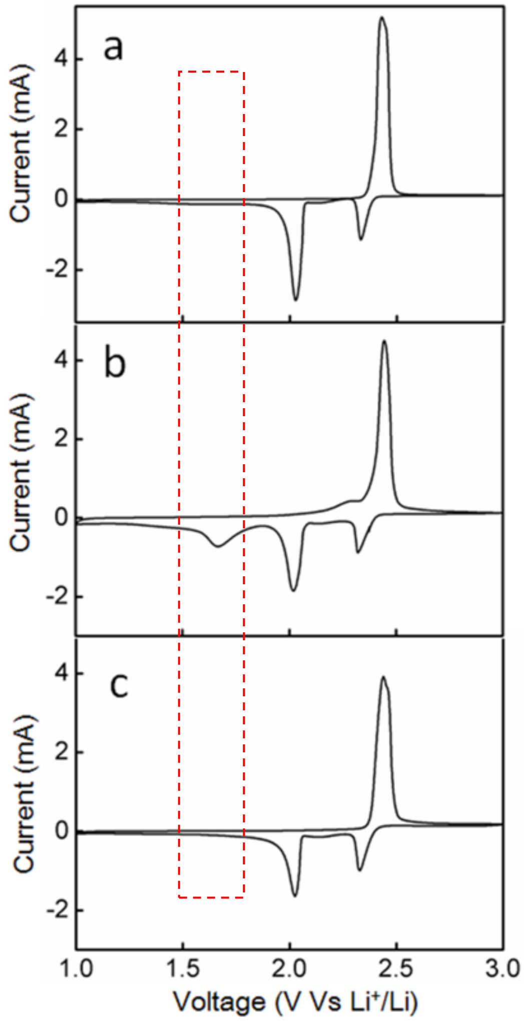

Supplementary Figure 6 | Cyclic voltammetry analysis. CV curves of different cells with **(a)** CMK-3/S, **(b)** CMK-3/S@PANS and **(c)** CMK-3/S@PANS@TPS cathodes.

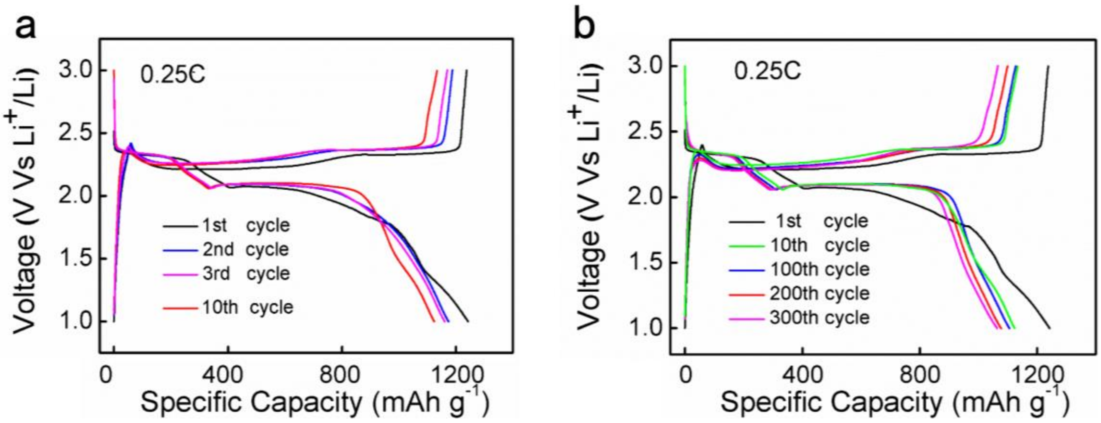

Supplementary Figure 7 | The discharge-charge curves at different cycles. Evolution of discharge-charge curves of CMK-3/S@PANS@TPS cathode **(a)** at the first 10 cycles and **(b)** during long cycles at 0.25C.

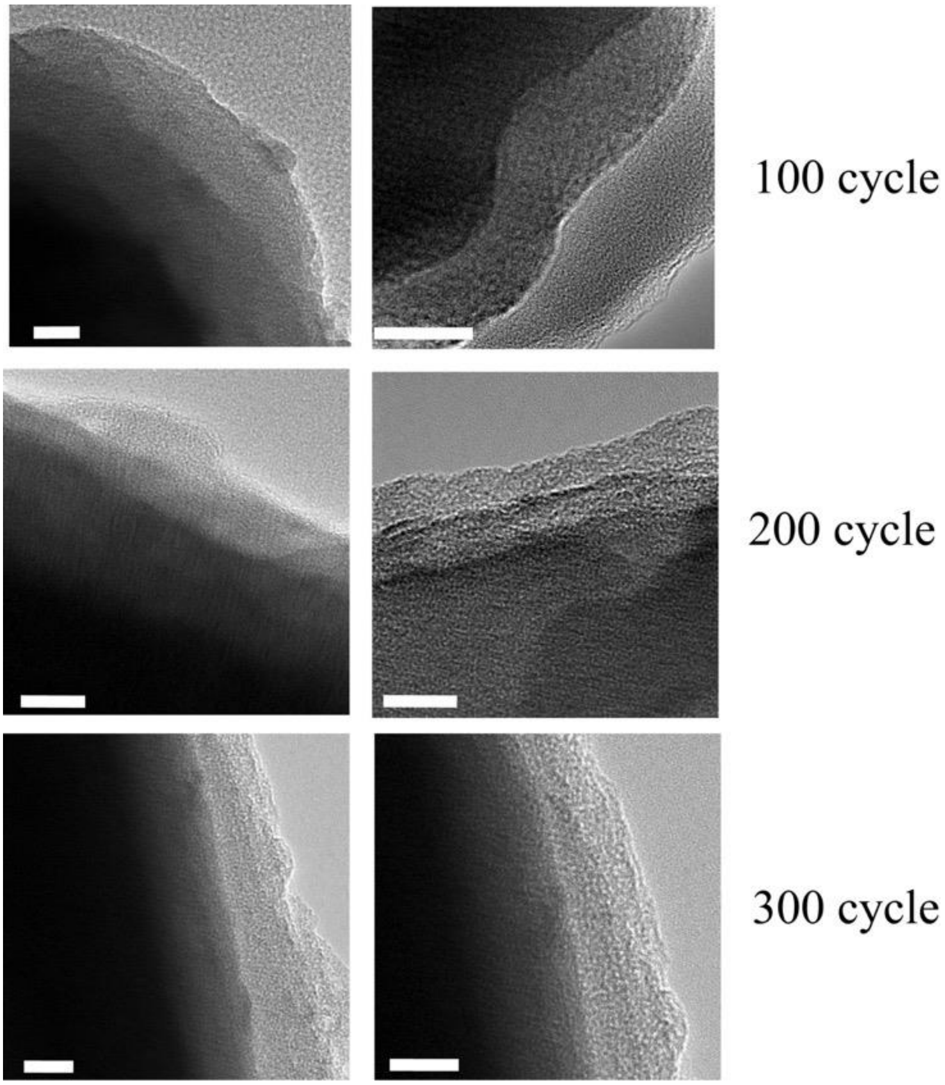

Supplementary Figure 8 | The TEM images of CMK-3/S@PANS@TPS after 100 , 200 and 300 cycles, all of the scale bars are 20 nm.

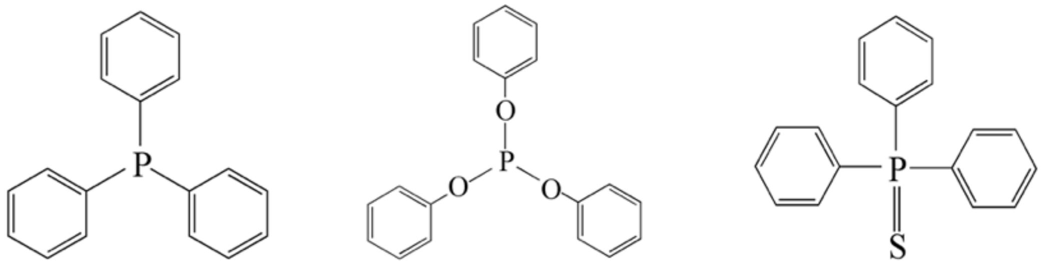

TPP TPPi TPS

Supplementary Figure 9 | The molecular structure of TPP, TPPi and TPS.

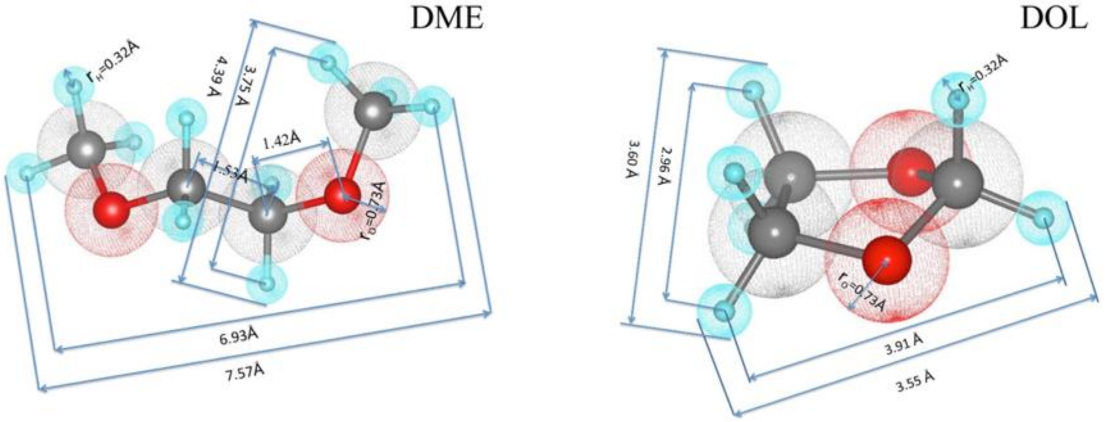

Supplementary Figure 10 | Theoretical calculation of the dimensions of DOL and DME molecules.

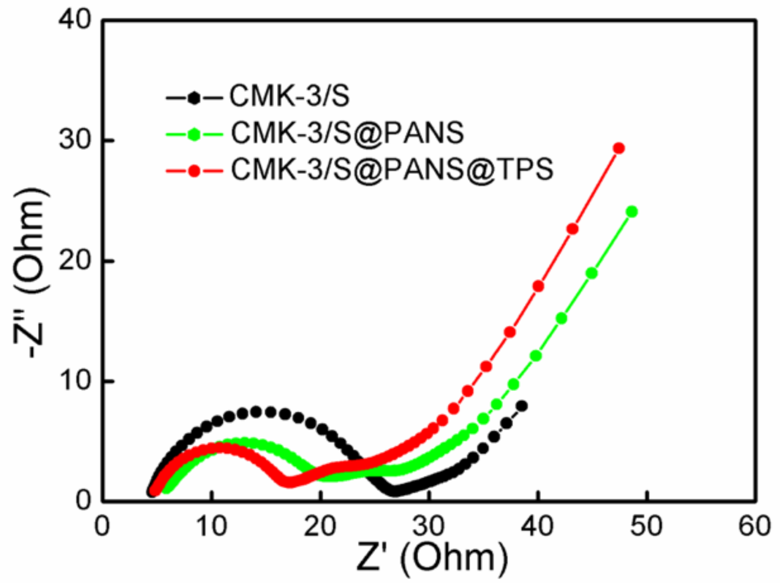

Supplementary Figure 11 | EIS of the cells with CMK-3/S, CMK-3/S@PANS and CMK-3/S@PANS@TPS cathodes at open circuit conditions.

Supplementary Tables

Supplementary Table 1. The XPS peaks of S_{2p} and P_{2p} of CMK-3/S, CMK-3/S@PANS and CMK-3/S@PANS@TPS

	**CMK-3/S**	**CMK-3/S@PANS**	**CMK-3/S@PANS@TPS**
S (2p)	$2p_{1/2}$： 165.3eV<br>$2p_{3/2}$： 164.1eV	$2p_{1/2}$： 165.3eV<br>$2p_{3/2}$： 164.1 eV	$2p_{1/2}$： 165.3eV<br>$2p_{3/2}$： 164.1 eV
		$2p_{1/2}$： 163.5 eV<br>$2p_{3/2}$： 162.3 eV	$2p_{1/2}$： 163.3eV<br>$2p_{3/2}$： 162.1 eV
P (2p)	--	--	132.7eV

Supplementary Notes

Supplementary Note 1. Reaction of triphenylphosphine and sulphur in the ether-based solvent

The reaction between TPP and sulphur in the ether-based electrolyte solvent is confirmed via multiple characterization methods. As illustrated in the Supplementary Fig. 2a and b, sulphur powder was added to a DOL/DME (1:1, v/v) solution containing TPP. The molar feed ratio of TPP to S was ~ 1.2:1 in order to ensure complete reaction of sulphur. After 3 hours of siting in room temperature, the yellow sulphur powder disappeared and the solution turned transparent and colorless. The ^{31}P NMR spectrum of the production solution shows the presence of both TPP and TPS (Supplementary Fig. 2c). The conversion ratio was not quantified because of the inaccuracy in feed ratio of the reactants in deuterated solvent for NMR measurements.

Under the same reaction condition, TPP was also mixed with the lithium salt used in the electrolyte, lithium bis(trifluoromethanesulphonyl)imide (LiTFSI). The NMR spectrum of the resulting solution shows no new peaks (Supplementary Fig. 2d), which indicates that no reaction occurs between LiTFSI and TPP.

Finally, to find out whether triphenylphosphine is oxidized during charge/discharge cycles, a cell with Al foil as working electrode and Li as the reference electrode and the counter electrode was assembled. LiTFSI/ in DOL/DME containing 2 wt% TPP was used as electrolyte. The cyclic voltammetry was measured between 1-3 V as shown in Supplementary Fig. 1e. No reduction or oxidation peaks are found in this voltage range, which means that triphenylphosphine does not get oxidized in this system.

Supplementary Note 2. Thermal gravimetric and derivative thermogravimetric analysis

As shown in the Supplementary Fig. 5a, the mass loss ratio of the CMK-3/S, CMK-3/S@PANS and CMK-3/S@PANS@TPS in the range of RT to 500 ^{o}C are 68.4%, 69.3% and 69.9%, respectively. This result is quite reasonable and detailed analysis is shown below.

Three new control samples including pure TPS, PANS and carbonized PAN (cPAN) are analyzed. The cPAN sample was prepared in the same way as the PANS except that no S powder was used (300 °C, 3 h). As shown in Supplementary Fig.5b, the DTG analysis of the pure CMK-3/S, TPS, PANS and cPAN show decomposition peaks at ~150-250 °C, ~250-350 °C, ~200-450 °C and ~300-500 °C, respectively. Supplementary Fig. 5c shows that the mass loss percentage of pure CMK-3/S, TPS, PANS and cPAN samples are 68.4%, 100%, 21.9% and 30.6%, respectively.

Therefore, the mass loss of the CMK-3/S@PANS@TPS sample can be understood in the following: the mass loss before ~300 °C can be attributed to the sulphur evaporation/decomposition, and the mass loss at 300-500 °C arises from the decomposition of TPS, PANS and PAN. Since the PANS and TPS wrapping layers are very thin layers, the weight of these layers in the total mass of the CMK-3/S@PANS@TPS materials is very small, ~2.0 wt% according to Supplementary Fig. 5d.

Supplementary Note 3. Cyclic voltammetry analysis.

Compared to the typical sulphur reduction peaks at 2.3 V and 2.1 V of CMK-3/S cathode,[4] the CMK-3/S@PANS material displays another reduction peak at 1.7V, which is related to the reduction of short sulphide chains linked to the PAN backbones.[5] In CMK-3/S@PANS@TPS cell, however, the peak at 1.7V almost disappears compared to the CMK-3/S@PANS cell. This confirms that the short-chain sulphide on PAN backbone is largely consumed in the reaction with TPP.

Supplementary Note 4. XPS characterization.

All XPS spectra were fitted with Shirley-type background by the software (XPS Peak). The fitting criterions of S are as follows: the position (1.18 eV between S $2p_{3/2}$ and S $2p_{1/2}$), the peak area ratio (S $2p_{3/2}$: S $2p_{1/2}$ = 2 : 1) and equal full width at half maximum.[6]

Supplementary References

1. Bartlett, P. D. & Meguerian, G. Reactions of Elemental Sulfur. I. The Uncatalyzed Reaction of Sulfur with Triarylphosphines1. *J. Am. Chem. Soc.* **78**, 3710–3715 (1956).
2. Chung, W. J. *et al*. Elemental Sulfur as a Reactive Medium for Gold Nanoparticles and Nanocomposite Materials. *Angew. Chem. Int. Ed.* **50**, 11409–11412 (2011).
3. Fanous, J., Wegner, M., Grimminger, J., Andresen, Ä. & Buchmeiser, M. R. Structure-Related Electrochemistry of Sulfur-Poly(acrylonitrile) Composite Cathode Materials for Rechargeable Lithium Batteries. *Chem. Mater.* **23**, 5024–5028 (2011).
4. Ji, X., Lee, K. T. & Nazar, L. F. A highly ordered nanostructured carbon-sulphur cathode for lithium-sulphur batteries. *Nat. Mater.* **8**, 500–506 (2009).
5. Zhang, S. S. Sulfurized Carbon: A Class of Cathode Materials for High Performance Lithium/Sulfur Batteries. *Fron. Energy Res.* **1**, 1–9 (2013).
6. Su, Y. S., Fu, Y., Cochell, T. & Manthiram, A. A strategic approach to recharging lithium-sulphur batteries for long cycle life. *Nat. Commun.* **4**, 2985–2993 (2013).
